# Supplementary material for: Patterns of Intron Gain and Loss in Fungi
Source: PLoS Biol. 2004 Nov 30;2(12):e422. doi: 10.1371/journal.pbio.0020422 (PMC532390; doi:10.1371/journal.pbio.0020422)
Supplement: Table S1 — Also available at http://genes.mit.edu/NielsenEtAl/. (4.3 MB ZIP). [file pbio.0020422.st001.zip › NielsenEtAl/html/87.html]

AN2174.1.NCU04370.1.MG01409.1.FG09689.1


```
 CLUSTAL W (1.82) Multiple Sequence Alignments - Introns Inserted


Sequence 1: NCU04370.1	1038 aa
Sequence 2: MG01409.1	1037 aa
Sequence 3: FG09689.1	1033 aa
Sequence 4: AN2174.1	1491 aa
Alignment Length: 1493 aa
Number Identitical Residues: 614 aa
Alignment Score (without introns) 28952


MG01409.1 	---~-------------------------------MA0DPSQGQVDL~VTRMQVDDS---
NCU04370.1	---~----------------------------MPISQ~DTQKNEVDL~VTRMQVDES---
FG09689.1 	---~-------------------------------MA0EQKQPEVDL~ATRMQVDES---
AN2174.1  	MAR0QGFHLHAFTITSSNLPVRDAAKLGKRRASAESE~TRPEKKAMT0DTKMQVDTPTQD
          	 :  ..    : : :::. .  .::. ..  : .       : :.    *:**** .:..

MG01409.1 	VVSAVATGEIDESLYSRQL~YVLGHEAMKRMGASNVLISGLKGLGVEIA~KNVALAGVKS
NCU04370.1	VAG--TTAEIDESLYSRQL~YVLGHEAMKRMSASNVLIVGLKGLGVEIA~KNVALAGVKS
FG09689.1 	VVG---NNEIDESLYSRQL~YVLGHEAMKRMGASNVLIVGLKGLGVEIA~KNIALAGVKS
AN2174.1  	TVEKIKQGDIDESLYSRQL2YVLGHEAMKRMGSSNVLVVGLKGLGVEIA1KNIALAGVKS
          	..      :********** ***********.:****: ********** **:*******

MG01409.1 	LSLHDPAPVAIADLSSQFFLRPEDVGKPRDQITAPRVAELNQYTPVKVHESAS2LTDDLS
NCU04370.1	LTLHDPEPAAWADLSAQFFLRPEDVGKPRDQVTAPRVAELNAYTPVHIHQSPS2LADNLS
FG09689.1 	LTLYDPAPVQIADLSSQFFLTPSDVGKPRDEVTVPRVAELNAYTPVKLHQSPG~LDGDLS
AN2174.1  	LTLYDPAPVAISDLSSQFFLQPQDVGKPRAEVTAPRVAELNSYVPVTIHEGSS~LVENLE
          	*:*:** *.  :***:**** *.****** ::*.******* *.** :*:... *  :*.

MG01409.1 	QLDKYQVVVLTNAPLVSQKAVGDYCHSKGIYFIAADTFGLFGALFCDFGEKFTVIDSTGE
NCU04370.1	QFDKYQVVVLTNQHTDLQTIVGEYCHSKGIYFIAVNTHGLFGGIFCDFGDNFTVMDTNGE
FG09689.1 	QFDKYQVVVLTNAPIHQQKAIADYCHSKGIYVVVADTFGLFGSVFCDFGEKFTVIDPTGE
AN2174.1  	QLKRYQAIVLTLTPLKEQLVIADFCHKNGIYLTIADTFGLFGYLFNDFGKNFTVGDSTGE
          	*:.:**.:***      *  :.::**.:***.  .:*.**** :* ***.:*** *..**

MG01409.1 	NPLNGMVNGIDEEGLVTTSDDARHGLEDGDYVTFTEVQGMEGLNGCQPRKITVKGPYTFS
NCU04370.1	NPVNGIVAGIDEEGLVSALDETRHGLEDGDYVTFSEVEGMEGLNGCEPRKVTVKGPYTFS
FG09689.1 	TPLSGIVAGIDEEGMVSALDETRHGLEDGDYVTFSEVEGMEALNGAEPRKITVKGPYTFS
AN2174.1  	DPVGGIVADISEDGLVSALDETRHGLEDGDFVTFTEVKGMEGLNNSAPRKVTVKGPYSFT
          	 *:.*:* .*.*:*:*:: *::********:***:**:***.**.. ***:******:*:

MG01409.1 	IGDVSGLGTYQRGGLYQQVKMPKIIDFKSFSESLADPEFVFSDYAKFDRPQQLHVGFQAL
NCU04370.1	IGDVSGLGQYKKGGLYQQVKMPKTIEFKSITNALKDPEFVISDFAKFDRPQQLHIGFQAL
FG09689.1 	IGDVSGLGQYKRGGMYQQVKMPKIINFKDFTTSLKEPEFLISDFAKFDRPQQLHLGFQAL
AN2174.1  	IGDVSGLGTYQGGGLFTQVKMPKFIDFEPLSEQIKKPEFLISDFAKFDRPQQLHIGVQAL
          	******** *: **:: ****** *:*: ::  : .***::**:**********:*.***

MG01409.1 	HAFAQTHGRLPRPMNAEDAIVVANSAKEFAKASGIEVEFDDKLLTELSYQASGDLNPMAA
NCU04370.1	HAFAKSQGRLPRPMNDEDALVVIASAKEFAKQQGVDVEFDDKLLKELSYQATGDLNPMAA
FG09689.1 	HAFQLTHKRLPNPMDDDDAIVVLGAAKTFAEQEGLEIELDEKLLKELSYQAQGDLNPMAA
AN2174.1  	HKFAETKGHFPRPHHESDAQELLQIANGLASSQEEKVELDEKLLKELSYQALGDLNPLAA
          	* *  :: ::*.* . .**  :   *: :*. .  .:*:*:***.****** *****:**

MG01409.1 	FFGGITAQEVLKAVSGKFHPVKQWLYFDSLESLPTNIPRTEELCQPINSRYDGQIAVFGK
NCU04370.1	FFGGLTAQEVLKAVSGKFHPIKQFMYFDALEALPTNSKRTEELCAPTGSRYDGQIAVFGK
FG09689.1 	YFGGLVAQEVLKAVSGKFQPIVQWMYFDSLESLPTSTKRSAELCKPTGSRYDGQIAVFGT
AN2174.1  	FFGGIVAQEVLKAVSGKFGPVHQWLYFDSLESLPTSVTRSEETCKPLGTRYDGQIAVFGK
          	:***:.************ *: *::***:**:***.  *: * * * .:**********.

MG01409.1 	AYQDKVANMKQFLVGAGAIGCEMLKNWAMIGLGTGPEGKIVITDMDSIEKSNLNRQFLFR
NCU04370.1	EFQEKISNVKQFLVGAGAIGCEMLKNWAMIGLGTGPEGKITVTDMDSIEKSNLNRQFLFR
FG09689.1 	EYQNKIANLKQFLVGAGAIGCEMLKNWAMIGLGTGPEGKIWVTDMDSIERSNLNRQFLFR
AN2174.1  	EFQDKIANLTQFLVGAGAIGCETLKNWAMMGLGTGPKGKIYVTDMDQIEKSNLNRQFLFR
          	 :*:*::*:.************ ******:******:*** :****.**:**********

MG01409.1 	AKDVGHMKSDCAAKAVQAMNPELEGHILCLKDRVSADTEHIFNEDFWNSLDGVTNALDNV
NCU04370.1	PKDVGQMKSDCAAKAAQAMNPDLEGHIVSLKDRVSPETEEIFNEEFWQGLDGVTNALDNV
FG09689.1 	ADDVGKMKSDRAALAVQRMNPDLEGHMITLKERVSADTESVFNEEFWHNLDGVTNALDNV
AN2174.1  	SKDVGKLKSECASAAAVAMNPELEGKIVTLKDRVGPDTEHIFNEEFWEGLDGVTNALDNV
          	..***::**: *: *.  ***:***::: **:**..:** :***:**..***********

MG01409.1 	EARTYVDRRCVFFHKSLLESGTLGTKGNTQVVLPNLTESYSSSQDPPEQSFPMCTLRSFP
NCU04370.1	EARTYVDRRCVFFHKPLLESGTLGTKGNTQVVLPRLTESYSSSQDPPEQSFPMCTLRSFP
FG09689.1 	EARTYVDRRCVFFQKPLLESGTLGTKGNTQVVLPHLTESYSSSQDPPEKEFPMCTIRSFP
AN2174.1  	EARTYVDRRCVFFRKPLLESGTLGTKGNTQVVLPRITESYSSSQDPPEKSFPMCTLKSFP
          	*************:*.******************.:************:.*****::***

MG01409.1 	NKIEHTIAWARE-LFDSSFIRPAETVNLYLTQPNYLESTLKQ-GNEKQTLEMLRDSLTKD
NCU04370.1	NKIEHTIAWARE-LFESSFVKPAETVNLYLTQPNYLDTTLKQSGNEKATLEMLADFLKHE
FG09689.1 	NKIDHTIAWSKEYMFEKLFVKAPQTVNLYLTQPQFIENSLKQGGNHKETLETIRNYLTTE
AN2174.1  	NRIEHTIAWARD-LFQTYFVGPPEAVNMYLSQPNYIEQTLKQAGNEKQTLEHLRDFLVTE
          	*:*:*****::: :*:. *: ..::**:**:**:::: :***.**.* *** : : *  :

MG01409.1 	RAMSFEDCVIWARGLFEKNYNNAIKQLLHNFPKDSVSSTGTPFWSGPKRAPDPLAFDPSN
NCU04370.1	RPLTFEDCVQWARMLFEKQYNNAIQQLLYNFPKDSVSSTGTPFWSGPKRAPDPLKFDPEN
FG09689.1 	RPRTFEDCIAWARQLFESEFSNKIQQLLYNFPKDSETSSGTPFWSGPKRAPDALKFDPNN
AN2174.1  	KPANFDDCIVWARNQFEAQYNNAIQQLLYNFPRDSTTSTGQPFWSGPKRAPTPLKFDSTN
          	:. .*:**: ***  ** ::.* *:***:***:** :*:* ********** .* **. *

MG01409.1 	PTHFMFVVAAANLHAFNYNINVKDKSKQDYLDVLSNMIIEEFEPDPTVRIQADEKEP0DP
NCU04370.1	PTHFSFLEAATNLHAFNYSINAKGKSKADYLRALEGMIVPDFSPDSNVKIQADEKEP0DP
FG09689.1 	PSHFGFIVAAANLHAFNYNIKSPGTDKSIYLRELENVIVPDFTPDSNVKIQADDKEP~--
AN2174.1  	PTHLGFIIAGANLHAYNYGIKNPGVDKGYYRKIVDNMIIPEFTPKSGVKIQASDNDP~DP
          	*:*: *: *.:****:**.*:  . .*  *   :..:*: :* *.. *:***.:::* ..

MG01409.1 	N~AGAG--AFDDTAEINNIVKELPSPKDLAGFKLTPVEFEKDDDTNYHIDFITAASNLRA
NCU04370.1	N~ADNT--AFDDESELGNLKSQLPEPKSLAGFKLNVVEFEKDDDTNYHIDFITAASNLRA
FG09689.1 	V0EAES--SFDDNDEIKKLADSLPSPSSLSGFQLVPVDFEKDDDSNHHIDFITACSNLRA
AN2174.1  	N~AEASGSSFDDNDEIKRLVEILPSPKSLEGFRLNPVEFEKDDDTNHHIDFITAASNLRA
          	      .::***  *: .: . **.*..* **:*  *:******:*:*******.*****

MG01409.1 	ENYKIEQADRHKTKFIAGKIIPAIATTTALVTGLVVFELYKIIDGKDDIEQYKNGFINLA
NCU04370.1	ENYKIEPADRHKTKFIAGKIIPAIATTTALVTGLVVLELYKIIDGKTDIEQYKNGFINLA
FG09689.1 	ENYKIEPADRHKTKFIAGKIIPAIATTTALVTGLVVLELYKIIDGKDDLEQYKNGFINLA
AN2174.1  	ENYEITPADRHKTKFIAGKIIPAIATTTALVTGLVALELLKIIDGKDDIEQYKNGFVNLA
          	***:*  ****************************.:** ****** *:*******:***

MG01409.1 	LPFFGFSEPIASPKVEYKGPNGEKVKLDKIWDRFEIGDVTLKELIDDFEKRGLSITMLSS
NCU04370.1	LPFFGFSEPIASPKVEYNGPNG-KVTLDKIWDRFEVGDITLQELIDDFEKRGLSISMLSS
FG09689.1 	LPFFGFSEPIASPKMEYQGPDG-KVKLDRIWDRFEIEDITLQELLDTFKAKGLTISMLSS
AN2174.1  	LPFFGFSEPIASPKTKYQGKQG-EVTIDQIWDRFEVDDIPLQDFLKHFSDLGLEISMVSS
          	************** :*:* :* :*.:*:******: *:.*::::. *.  ** *:*:**

MG01409.1 	GVSLLYASFFPPAKLKERYSMK~LSQLVEHISKKPIPEHQKEVIFEVVTEDADGEDVEVP
NCU04370.1	GVSLLYASFFPPAKLKDRYTLK~LSELVETISKKPIPAHQKELIFEVVTEDADGEDVEVP
FG09689.1 	GVSLLYASFFPPSKLKERYALK~LSQLVETISKKPIPAHQKDVIFEIVAEDLNEEDVEVP
AN2174.1  	GVSLLYASFYGPSKVKDRLPMK2MSELVEHISKKPIPDHQKNVIFEVTAEDQTEQDVEIP
          	*********: *:*:*:* .:* :*:*** ******* ***::***:.:**   :***:*

MG01409.1 	~YIKVKIR----------------------------------------------------
NCU04370.1	~YIKARIR----------------------------------------------------
FG09689.1 	~YIKVKVA----------------------------------------------------
AN2174.1  	2AKSGLVRLSDPSVINVISCEGLQAPPQSSASFTHINLVLFLALIFMFLVLRRANQLVQH
          	   .  :  :..:  .  :... .:...:::: :  .     :          :..  . 

MG01409.1 	------------------------------~-----------------------------
NCU04370.1	------------------------------~-----------------------------
FG09689.1 	------------------------------~-----------------------------
AN2174.1  	LSPFSALFGPSNSNISRNLSPALLAKREMA0TDISKKITDWVNPGDKSGEFKRQASVFRN
          	 :. ::  ..:.:. : . :.:  :. . : :. :.. :.  .....:.. . .::   .

MG01409.1 	----------------------------~-----------------------------~-
NCU04370.1	----------------------------~-----------------------------~-
FG09689.1 	----------------------------~-----------------------------~-
AN2174.1  	LISKEAGAEFPPEKDRYHLYVSYACPWA1HRTLIVRKLKGLEDIISYTSVHWHLGEKG1W
          	  :..:.:. .....      : :.. :   :    . .. ..  : ::     ....  

MG01409.1 	------------------------------------------------------------
NCU04370.1	------------------------------------------------------------
FG09689.1 	------------------------------------------------------------
AN2174.1  	RFATPDEDIPGANTTPDPVHSSYSHLRDIYFANEPNYTGRFTVPVLYDKKTKRIVSNESS
          	  ::.... ..:.::...  :: :   .   :.... :.  : .   ...:.   :..::

MG01409.1 	------------------------------------------------------------
NCU04370.1	------------------------------------------------------------
FG09689.1 	------------------------------------------------------------
AN2174.1  	EIIRMFYYEFDDLLPAQYQKVDLFPPHLREQIDATNDWVYNDVNNGVYKSGFATTQEAYE
          	.       . ..  .:. .. .  ..   .. .::..   .. ...  .:. :::..: .

MG01409.1 	------------------------------------------------------------
NCU04370.1	------------------------------------------------------------
FG09689.1 	------------------------------------------------------------
AN2174.1  	RNVTTLFASLDRIEKHLADSKSAYFFGDDITEADIRLFTTIVRFDPVYVQHFKCNIRDIR
          	 . ::  :: .  ..  :.:.::   ... :.:.    ::    ..   .  ...  .  

MG01409.1 	------------------------------------------------------------
NCU04370.1	------------------------------------------------------------
FG09689.1 	------------------------------------------------------------
AN2174.1  	SGYPAIHAWLRRLYWDVPAFRETTQFEHIKKHYTKSHSQINPFGITPVGPTPDILPKDKE
          	:. .:  :       . .:  .::. .  ..  :.: :. .. . :. ..:..  .....

MG01409.1 	------
NCU04370.1	------
FG09689.1 	------
AN2174.1  	VNAVKH
          	 .: .
```
